# High-Efficiency Electromagnetic Translational–Rotary Harvester for Human Motion Impact Energy

**DOI:** 10.3390/s25113453

**Published:** 2025-05-30

**Authors:** Shuxian Wang, Shiyou Liu, Zhiyi Wu

**Affiliations:** 1 College of Integrated Circuits, Chongqing University of Posts and Telecommunications, Chongqing 400065, China; wangsx@cqupt.edu.cn; 2 School of Computer, Chongqing University, Chongqing 400038, China; shiyouliu@163.com; 3 Chongqing Qingshan Industry Co., Ltd., Chongqing 402761, China; 4 International Frontier Interdisciplinary Science Research Institute, Beihang University, Beijing 100191, China

**Keywords:** energy harvester, motion impact energy, electromagnetic induction, translational–rotary harvester, wearable electronics

## Abstract

This paper presents an electromagnetic translational–rotary motion impact energy harvester based on a magnetic cylinder rotated around a fixed magnetic ring. It is beneficial for capturing impact energy generated by natural human motions, such as clapping, boxing, and stomping. The energy harvester consists of a circular housing, twelve coils, a magnetic cylinder, and a magnetic ring. Once activated, the magnetic cylinder revolves and rotates around the magnetic ring, inducing a significantly large electromotive force across the twelve coils. According to Faraday’s law, the output voltage generated by the coils is proportional to the turns, enabling the efficient harvesting of biomechanical waste energy. Moreover, the energy harvester can convert translational motion from any orientation into a multi-circle rotational motion of the low-damping magnetic cylinder, which passes through twelve coils and applies a variable magnetic field across them. During a single excitation event, the prototype harvester was able to charge a 470 μF, 25 V capacitor to over 0.81 V in just 39.5 ms. The energy output and effective average power were calculated to exceed 0.15 mJ and 3.80 mW, respectively.

## 1. Introduction

Recently, various strategies have been developed to extend the operating time of batteries in human motion devices, wireless electrical equipment, and biomedical devices, with the ultimate goal of achieving self-powered wearable electronics. Differential transduction principles, including electromagnetic [1,2,3], piezoelectric [4,5,6], and triboelectric effects [7,8,9], have been employed as key mechanisms. Among these, triboelectric nanogenerators have shown numerous appealing advantages, such as exceptionally high output voltage, low cost, remarkable structural design versatility, exceptional stability and durability, and environmental friendliness [10,11]. However, friction has two opposing effects on the triboelectric performance: while it initially enhances output, prolonged friction reduces the efficiency of triboelectric nanogenerators. Despite this limitation, triboelectric nanogenerators remain valuable for developing flexible devices. In contrast, the electromagnetic effect is the most common method in terms of output performance as it generally provides sufficient power output [12].

The most popular technique for capturing vibration energy is through resonant mechanical devices using spring-mass dampers. However, a resonant system’s optimal operating condition is limited by its narrow bandwidth, which is only achieved when the external vibration matches the system’s resonant frequency [13,14]. To address this issue, researchers have explored electromagnetic energy harvester (EEH) designs by utilizing a translational motion levitating magnet as a springless proof mass [15,16,17]. Although various designs for non-resonant energy harvesters have been proposed [18], many of them still face challenges. For example, energy from vibration, rotation, and even human motion has been successfully harvested using a motion-levitating magnet inside a coil-based tube. However, when the harvester is tilted or rotated in certain orientations, its output significantly decreases [19]. This suggests that the harvester can only operate optimally in specific conditions, making it difficult to gather consistent energy. Some designs show that reducing power consumption for low-power sensing helps ease the energy harvesting process [20,21]. This leads to the development of a symmetrical 2-dimensional harvester that can be operated in any plane orientation [22,23]. Nonetheless, the limited degrees of freedom in such designs make them unsuitable for motion energy harvesting. Recently, hybrid translational–rotary harvesting techniques have gained attention due to their high-power density [24,25,26]. For instance, Kuang and Zhu employed repulsive and attractive magnetic forces to create a parametrically excited nonlinear magnetic pendulum, which can effectively capture vibration energy across multiple frequency ranges, though precise control of parameters is necessary [27]. Most devices use spheres to harvest energy from their surroundings. A magnetic sphere, which directly supplies a varying magnetic field to the coils, is considered optimal for electromagnetic energy harvesters [28,29]. However, due to inherent unpredictability, a single magnetic sphere cannot consistently produce a strong magnetic field for the coils [30]. To overcome this limitation, the magnetic sphere is connected to a magnetic cylinder beneath it, restricting its motion to a cycloidal path. The magnetic sphere is encased in a shell with a non-uniform mass distribution, allowing it to vibrate like a tumbler [31]. The eccentric mass, aided by gravity, facilitates creative motion from a single anchor point. Energy collectors utilizing eccentric mass-driven rotors and low-frequency excitations have been developed, but these designs are often complex [32,33]. Human motions such as stomping, boxing, and clapping are common in daily life, and the impact energy from these actions presents a valuable opportunity for energy harvesting. The design in Ref. [34] is capable of collecting mechanical energy at low frequencies (1 Hz), but the use of inertial systems and gears increases design complexity, which, in turn, raises manufacturing and maintenance costs. One promising approach involves quasi-zero-stiffness (QZS) mechanisms, which provide both low dynamic stiffness for resonance amplification and structural stability. For example, Liu et al. [35,36,37] investigated the nonlinear dynamics of a magnetic vibration isolator with higher-order stable QZS characteristics, revealing its potential to improve low-frequency energy conversion efficiency.

The energy harvester presented in this study is a hybrid translational–rotary device that captures the impact energy of human motion by rotating a magnetic cylinder around a fixed magnetic ring. This harvester converts translational motion from any orientation into multi-circle rotational motion of the low-damping magnetic cylinder, which passes through twelve coils, generating notably high electromotive forces. The motion of the magnetic cylinder has been demonstrated, and typical output waveforms of the harvester have been evaluated. Impact energy from actions such as clapping, punching, and stomping was successfully captured. The energy and effective average power generated by the harvester under each excitation have been calculated. In conclusion, this device has potential applications in wearable technology, toys, and other smart gadgets.

## 2. Analysis and Design of the Harvester

### 2.1. Harvester Design

**Figure 1:**
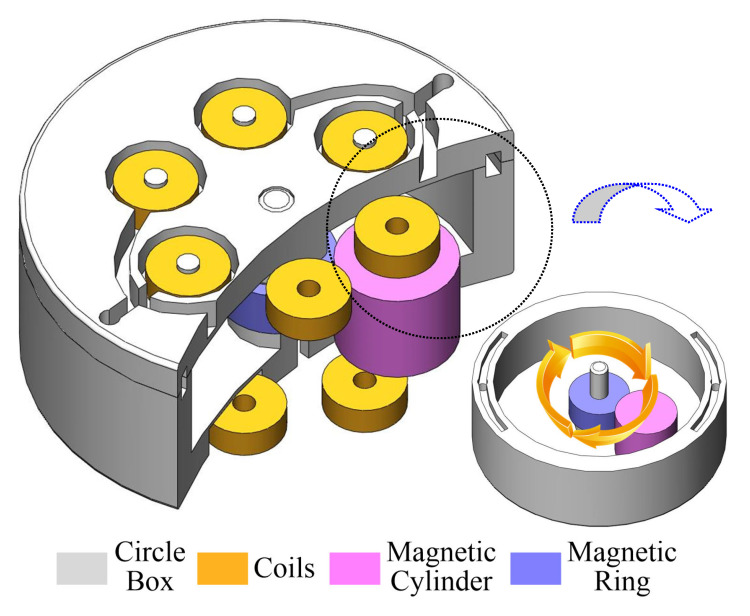
(Color online) Broken-out section and inside views of the motion impact energy harvester show the magnetic cylinder rotated around a fixed magnetic ring located in the circle box and the twelve coils placed in the box’s two ends, respectively.

The energy harvester consists of a circular housing, coils, a magnetic cylinder, and a magnetic ring. Figure 1 shows a cutaway section and internal views of the motion impact energy harvester. The magnetic attraction forces between the magnets are concentrated at both ends, ensuring that the magnetic cylinder is held close to the magnetic ring. At the end of the housing, the coils are divided into upper and lower sets. Each set includes six coil windings connected in series, while the upper and lower coil sets are connected in parallel. To more efficiently detect changes in the magnetic field, the angle between adjacent coils in the two layers is 30°. When the harvester is excited by an impact, the magnetic cylinder rotates around the magnetic ring while also spinning. According to Faraday’s law, the coils will detect a voltage signal. Compared to previous designs [38], our low-damping magnetic cylinder design allows for longer motion duration, effectively extending the energy harvesting process and improving overall energy harvesting efficiency. In contrast, earlier designs with bearing structures may suffer performance degradation over time due to friction or contact fatigue.

### 2.2. Working Principle

When the motion impacts the energy harvester, triggered by the wearer’s natural movements, the magnetic cylinder revolves around and rotates along the magnetic ring, inducing a significantly large electromotive force across the twelve coils. According to Faraday’s law, the coils generate an output voltage proportional to the number of turns, enabling the harvesting of waste biomechanical energy. Additionally, there are no requirements for a specific initial alignment between the movers and the coils, allowing the motion impact energy harvester to be worn without any particular considerations or adjustments.

**Figure 2:**
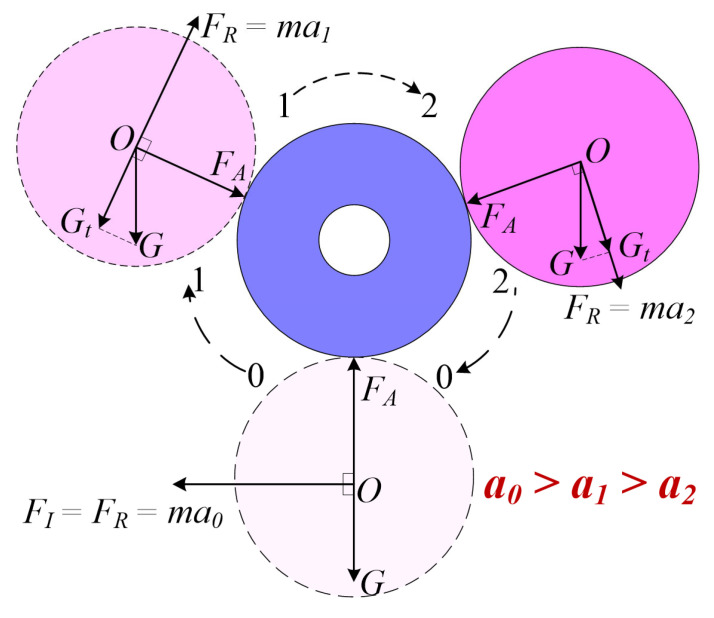
(Color online) A mechanical analysis diagram of the magnetic cylinder.

**Figure 3:**
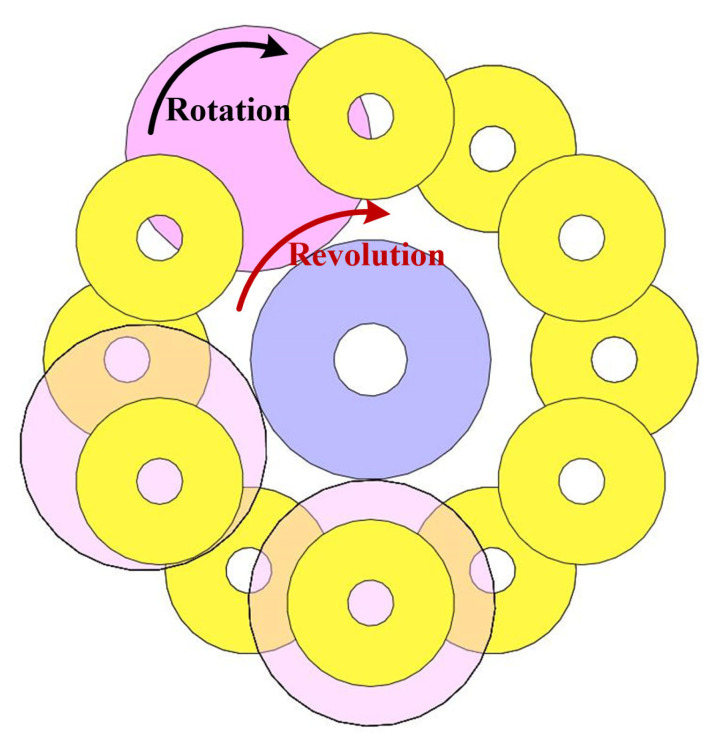
(Color online) A schematic of the working principle.

Figure 2 shows the mechanics of the vertically positioned motion impact energy harvester. In its resting state, the magnetic cylinder remains balanced under the influence of gravity (*G*) and the attractive force (*F_A_*) without any external excitation. Excitation from any direction disrupts this balance, except for extreme excitation along the vertical axis, which is unlikely to occur during human motion. For instance, consider a horizontal-left impulse excitation (*F_I_*), denoted as State-0. *F_I_* provides an initial spin acceleration (*a*_0_) to the magnetic cylinder. Under the influence of the attractive force, the magnetic cylinder begins spinning around the circumference of the magnetic ring. In the climbing phase (State-1), gravity acts as a resistance to the spin. A strong enough *F_I_* allows the magnetic cylinder to reach the descending phase (State-2), during which gravity aids in accelerating the spin. A sufficiently strong *F_I_* can cause the magnetic cylinder to spin several times before eventually oscillating at the balance point for a short duration. The relationship of the spin accelerations in these three states is *a*_0_ > *a*_1_ > *a*_2_. Thus, once an external force displaces the magnetic cylinder against the inner surface, the curved structure naturally guides it into a rolling trajectory along the magnetic ring. This results in a coupled revolution and self-rotation, passing through multiple coils and inducing voltage. The omnidirectional adaptability is ensured by the symmetric mechanical design and the low-damping nature of the magnetic cylinder support. When the device is excited by motion, the magnetic cylinder changes states. In addition to its orbital motion around the magnetic ring, the magnetic cylinder also undergoes continuous rotation along its central axis, as illustrated in Figure 3 This dual motion, combining revolution around the ring and axial spinning, creates a dynamic interaction with the surrounding coils. As the cylinder moves and rotates, the orientation and strength of its magnetic field fluctuate periodically. These variations induce a time-varying magnetic flux through the coil windings, thereby generating an alternating electromotive force according to Faraday’s law of electromagnetic induction.

**Figure 4:**
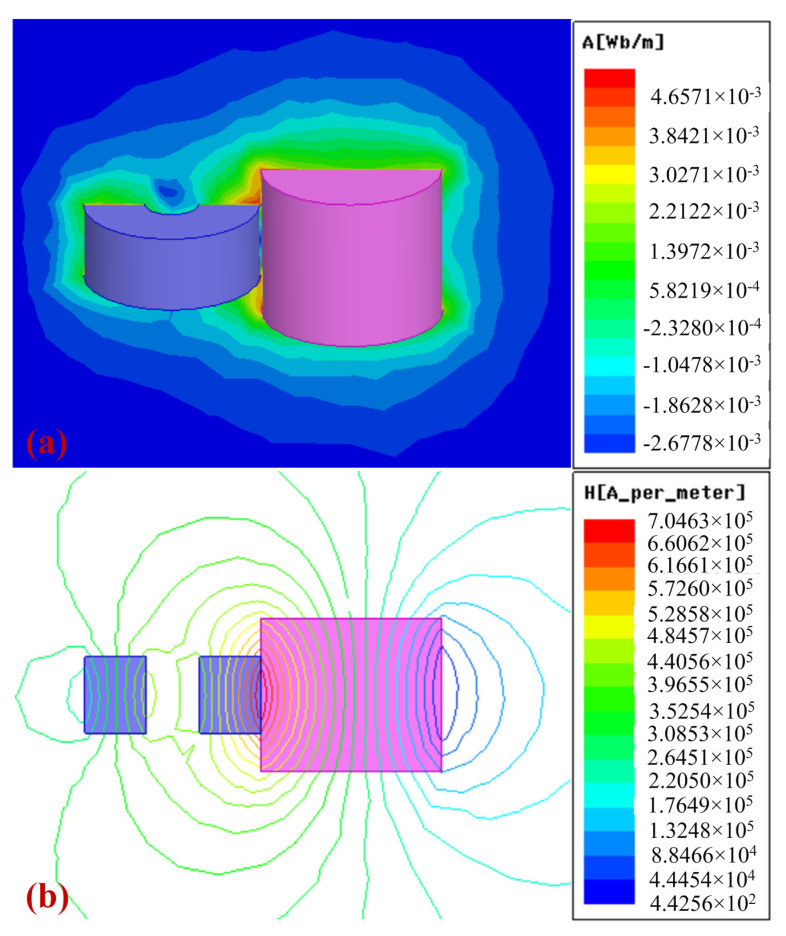
Magnetic simulation results of the motion impact energy harvester using Maxwell. (**a**) The magnetic cloud map of the magnetic cylinder and the magnetic ring. (**b**) The magnetic flux lines around the magnetic cylinder and the magnetic ring.

Due to the complexity of the analytical formula for the magnetic field produced by the interaction between the magnetic ring and the magnetic cylinder, the magnetic field distribution of the motion impact energy harvester is analyzed using Maxwell 16.0 software, as shown in Figure 4. Figure 4a displays the magnetic field cloud map where the magnetic field strength is primarily concentrated between the magnetic cylinder and the magnetic ring. Additionally, Figure 4b shows the magnetic force lines of the motion impact energy harvester. Both the magnetic cloud map and the magnetic force line distribution indicate that the magnetic cylinder is attracted to the magnetic ring due to the magnetic forces between them. This illustrates that the magnetic field strength between the two components is stronger than the surrounding magnetic field.

**Figure 5:**
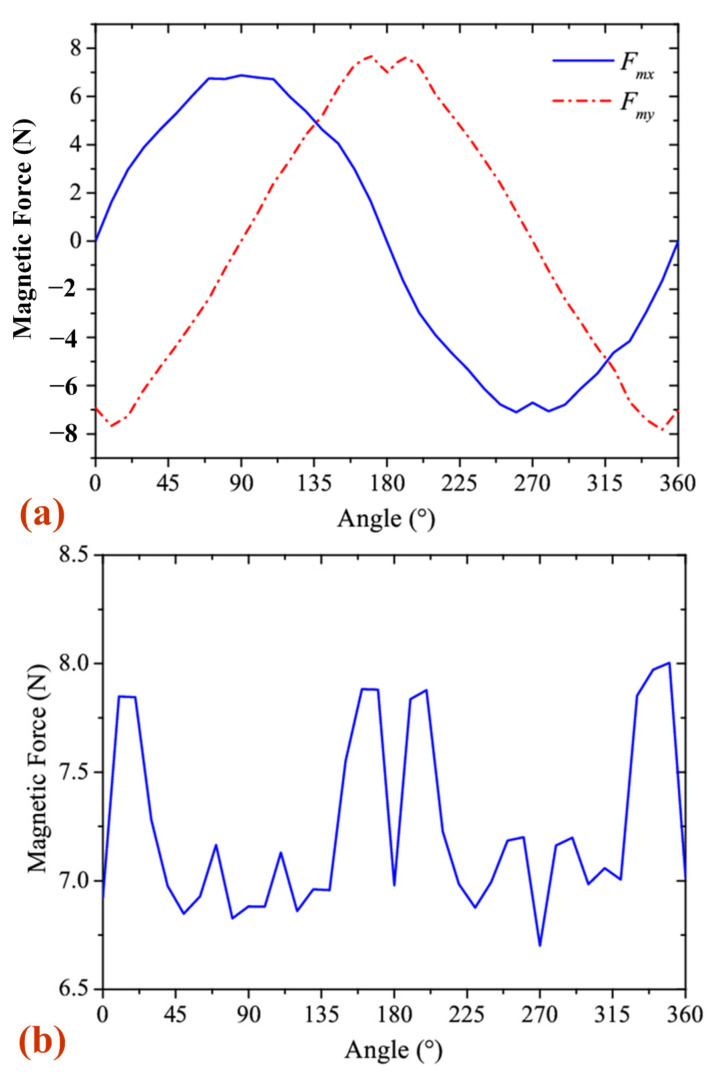
The magnetic force of the magnetic cylinder: (**a**) the component along the *x* and *y* direction; (**b**) the centripetal magnetic force.

As the magnetic cylinder rotates around the magnetic ring, the relationship between the magnetic force and the angle was generated numerically through electromagnetic simulation using Maxwell 16.0 software, as shown in Figure 5. Figure 5a shows that the magnetic force components along the *x* and *y* directions both exhibit a sinusoidal behavior, with a 90° phase difference between them. Additionally, at various angles, the centripetal magnetic force remains greater than 7 N, which is stronger than the gravitational force acting on the magnetic cylinder. Figure 5b illustrates how the magnetic force varies as the magnetic cylinder completes a full rotation. By adjusting the dimensions of the magnetic cylinder and the magnetic ring, the centripetal magnetic force can be tuned to reduce the magnetic damping of the magnetic cylinder.

**Figure 6:**
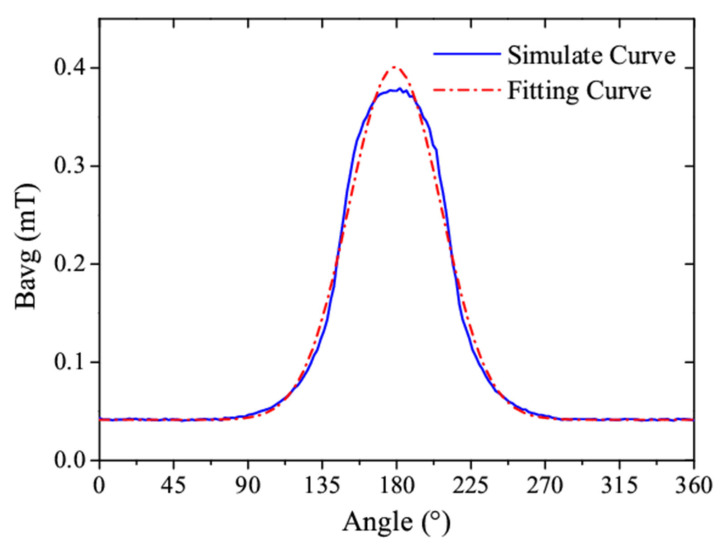
(Color online) A comparison between the induced voltage obtained from the finite element simulation and the analytical model derived from a mathematical model.

With the revolution of the magnetic cylinder, it applies the simulated output voltage and the analytical prediction, as shown in Figure 6. The close agreement between the two curves demonstrates that the simplified model can reasonably estimate the induced voltage under typical operating conditions. The variable magnetic induction intensity of the coil is a function of the angle, denoted as *B_avg_*(*θ*). A Gaussian function can be used to approximate it:

(1) $$B_{avg}\left(\theta \right)=0.041+0.359\times \exp(-0.5\times {\left(\frac{\theta -179.03}{27.894}\right)}^{2})$$

Thus, the fitting curve based on this mathematical model is consistent with the simulated curve, as shown in Figure 6.

According to the electromagnetic induction principle, the output voltage of the coil can be expressed as

(2) $$\begin{array}{ll} V_{out} & =-\frac{d\Phi }{dt}=-NS\frac{dB_{avg}\left(\theta \right)}{dt}\\ & =-NS\frac{dB_{avg}\left(\theta \right)}{d\theta }\frac{d\theta }{dt}\\ & =-NS\frac{dB_{avg}\left(\theta \right)}{d\theta }v(\theta ) \end{array}$$

where *N* and *S* are the coil’s turn number and magnetic flux area, respectively. *v*(*θ*) is the rotational speed of the magnetic cylinder.

From Equation (1), the derivative of *B_avg_*(*θ*) can be obtained as

(3) $$\frac{dB_{avg}\left(\theta \right)}{d\theta }=-0.00046\times (\theta -179.03)\;\times \exp(-0.5\times {\left(\frac{\theta -179.03}{27.894}\right)}^{2})$$

**Figure 7:**
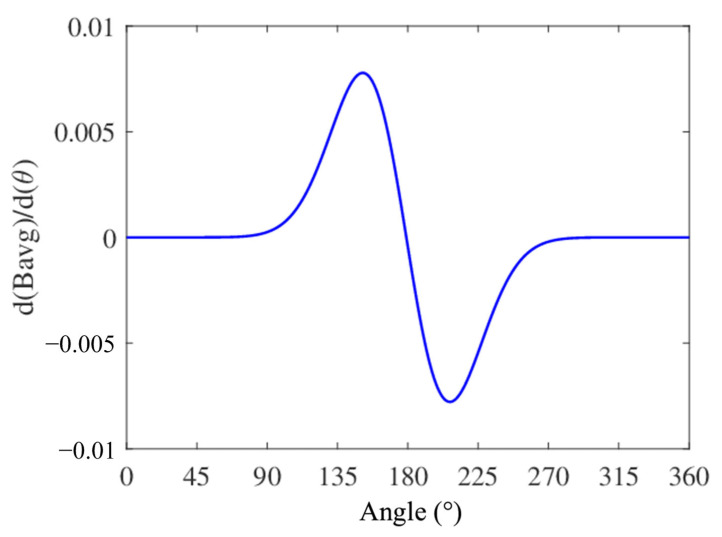
(Color online) The relationship between the derivative of *B_avg_*(*θ*) and the angle.

The values of the derivative of *B_avg_*(*θ*) under different *θ* are plotted in Figure 7.

At the same time, the rotational speed of a magnetic cylinder can be written as

(4) $$v\left(\theta \right)=\int_{0}^{t}adt$$

As the magnetic cylinder revolves around the magnetic ring, its acceleration gradually decreases under a single excitation, as shown in Figure 2. Consequently, *v*(*θ*) also decreases over time. By substituting Equations (3) and (4) into Equation (2), it can be observed that the output waveform of the coil retains the same shape, as shown in Figure 6, but exhibits damping characteristics. Additionally, due to the coil arrangement, the output waveforms of the coils have a phase difference, ensuring that the motion impact energy harvester maintains good output performance.

## 3. Results and Discussion

### 3.1. Establish Experimental Platforms

The harvester is fabricated based on the principle of electromagnetic energy conversion. A circular box (48 mm outer diameter, 19 mm height) contains 12 evenly distributed grooves and pillars on its ends, designed to hold 12 self-adhesive coils (2.2 mm inner diameter, 8 mm outer diameter, 2.9 mm height, ~13 Ω). A central pillar secures a magnetic ring (NdFeB, N38, 3.5 mm inner diameter, 11.5 mm outer diameter, 5 mm height). The box also features a circular cavity (40 mm outer diameter, 11 mm height) that provides motion space for a magnetic cylinder (NdFeB, N38, 11.8 mm diameter, 10 mm height), along with additional grooves and holes for the coil leads. The box is made from stereolithography material (DSM Functional Materials, Elgin, United States) via 3D printing.

**Figure 8:**
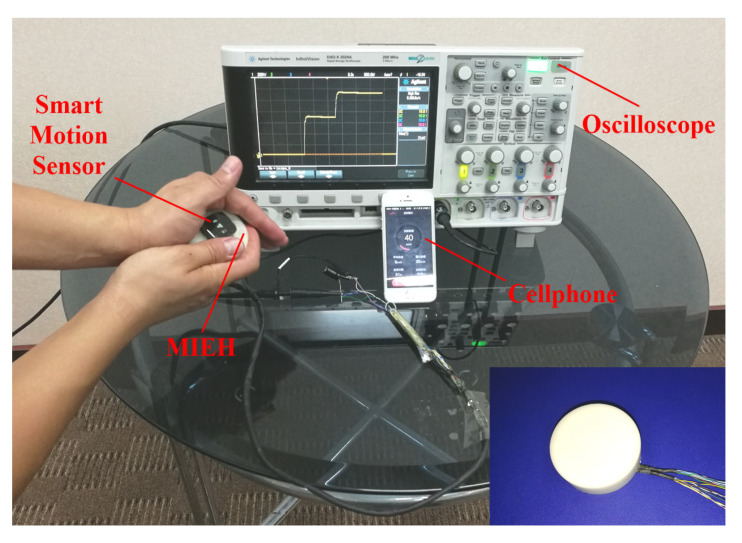
An experimental system and a photograph of the prototype motion impact energy harvester.

The device was designed and tested based on the theoretical analysis and design principles of the motion impact energy harvester. A photograph of the prototype, with an outer diameter of 48 mm and a height of 19 mm, is shown in the inset of Figure 8. The volume of the prototype is approximately 34.38 cm^3^. To evaluate its output performance, an experimental setup was established, as shown in Figure 8. An oscilloscope (InfiniiVision DSO-X2024A Digital Storage Oscilloscope, Agilent Technologies, Santa Clara, CA, USA) was used to monitor the typical output waveforms and the charge–discharge characteristics of the magnetic cylinder. Additionally, to estimate the impact velocities during typical human motions, a smart motion sensor (UnSense, Shenzhen UBC Technologies Co., Ltd., Shenzhen, China) was affixed to the moving body or harvester housing. The device includes a built-in IMU (inertial measurement unit) that measures acceleration and displacement to calculate approximate speeds. The velocity data are transmitted to a mobile device via Bluetooth and displayed in real time using the manufacturer’s app, as illustrated in Figure 8.

### 3.2. Output Characterization from Human Motion

**Figure 9:**
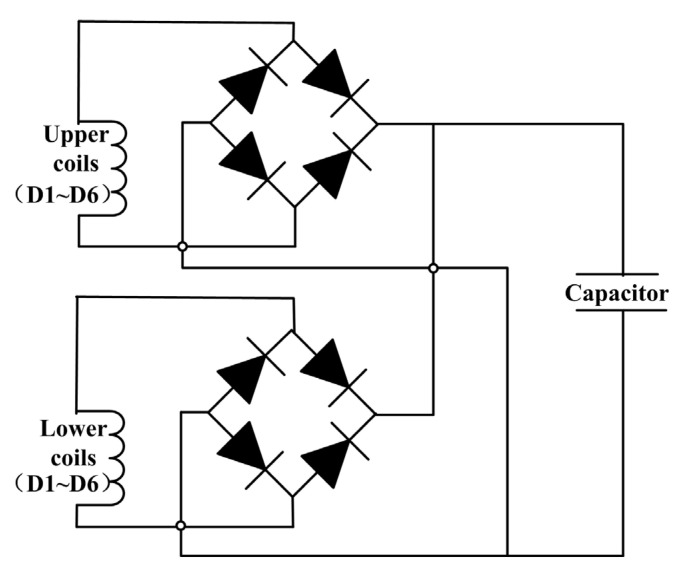
A circuit diagram of the harvester and a charging circuit for the capacitor.

The charging performance of the motion impact energy harvester was evaluated using typical signal tests involving human motion. In everyday life, motions such as clapping, boxing, and stomping are common, and the impact energy from these movements is harvested by the device. The power management circuit, which consisted of a simple rectification circuit and a capacitor, is illustrated in Figure 9. The twelve coils are divided into two groups: the upper six coils and the lower six coils. Each group shares a bridge rectifier, and the outputs of these two rectifiers are then connected in parallel to a common energy storage capacitor. This dual-group rectification approach balances simplicity and efficiency, minimizing mismatched back electromotive forces (EMF) among coils while reducing the number of diodes and associated conduction losses compared to individual rectification. The harvester was used to charge a 470 μF, 25 V capacitor, which was selected to match the typical voltage output characteristics of the harvester while providing a measurable voltage rise and avoiding early saturation, ensuring meaningful energy evaluation. In the following sections, the experimental results for clap, boxing, and stomping excitations will be presented.

#### 3.2.1. Clapping Test

**Figure 10:**
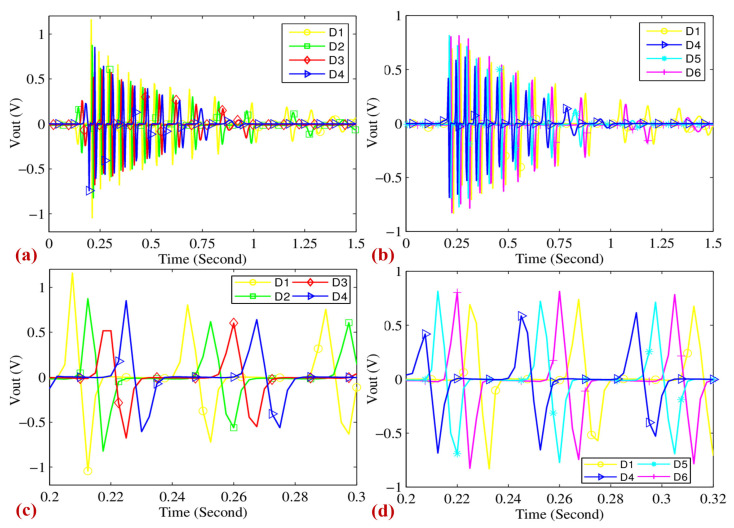
(**a**,**b**) The typical output waveforms of the motion impact energy harvester; D1~D6 are the coils in the same layer and are numbered in the counterclockwise direction. (**c**,**d**) The details of the output waveforms.

When the motion impact energy harvester is excited by changes in the surrounding environment, the device generates voltage. For example, during clap excitation, typical output waveforms of the harvester were tested by holding the device in one hand and striking it with the other. Coils D1 to D6, positioned in the same layer and numbered counterclockwise, exhibited synchronized behavior, indicating uniform magnetic field interaction. Since the oscilloscope only has four channels, the output waveforms of D1 to D6 were recorded in two sets, as shown in Figure 10. Figure 10a,b demonstrates that the output waveforms of the harvester exhibit a damped oscillation pattern, with the magnetic cylinder spinning at least 10 times after a single excitation. The amplitude of these waveforms varies significantly, with a maximum voltage of up to 1.2 V. Details of the output waveforms are shown in Figure 10c,d, and their shapes are consistent with those depicted in Figure 7, confirming that theory and practice align. This highlights the transient nature of the energy conversion process and underscores the importance of coil design and positioning for optimal energy capture. As the magnetic cylinder rotates, coils D1 to D6 reach their voltage peaks sequentially, corresponding to their physical arrangement. This ensures that the energy harvester consistently produces high-output waveforms throughout its operation, which is crucial for its performance.

**Figure 11:**
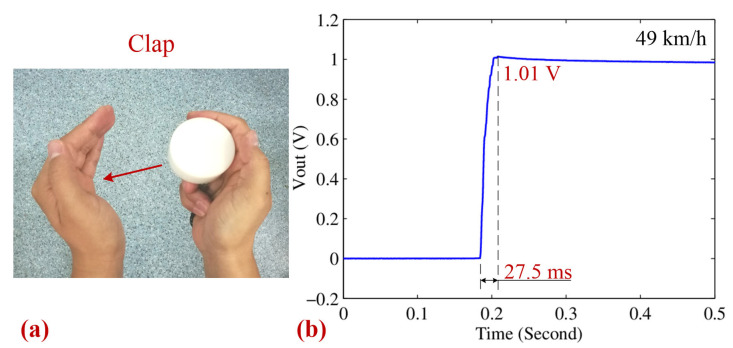
(**a**) Photographs of the clap excitation type. (**b**) The corresponding charging–discharging curves of a 470 μF 25 V capacitor for the clap type.

The clap excitation setup is illustrated in Figure 11a, where the motion impact energy harvester is firmly held in the right hand and used to pat the left hand. This setup effectively demonstrates the device’s capability to harness energy from simple, natural hand movements, making it highly relevant for wearable and portable energy-harvesting applications. The corresponding charge–discharge curves of a 470 μF, 25 V capacitor are shown in Figure 11b, highlighting the rapid energy accumulation process. The impact speed in this test reaches approximately 49 km/h, and within just 27.5 ms, the capacitor voltage rises to 1.01 V, storing over 0.24 mJ of energy. This results in an effective average power output of 8.72 mW, which is promising for low-power electronics, such as self-powered sensors and health-monitoring devices. The efficiency of this energy conversion is primarily attributed to the optimized design of the harvester, which ensures effective kinetic energy transfer during impact. Factors such as the alignment of the device, the mechanical properties of the contact surface, and the stability of the applied force play a crucial role in determining performance. Furthermore, the repeatability of energy harvesting through hand claps suggests that this method could be leveraged for practical applications where frequent hand movements occur, such as in interactive devices, smart wearables, or gesture-based energy harvesting systems.

#### 3.2.2. Boxing Test

**Figure 12:**
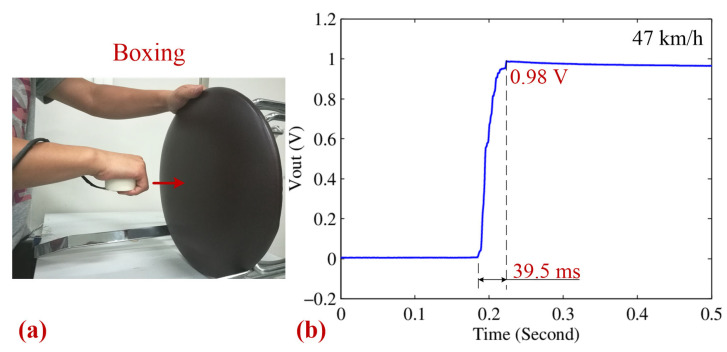
(**a**) A photograph of the boxing type. (**b**) The corresponding charging–discharging curves of a 470 μF 25 V capacitor for the boxing type.

To simulate a more vigorous activity, such as boxing, the motion impact energy harvester was firmly held in hand and repeatedly used to strike a rigid stool surface, as illustrated in Figure 12a. This experiment aimed to evaluate the harvester’s response under dynamic, high-intensity impact conditions, similar to those encountered in real-world scenarios. Figure 12b presents the corresponding charge–discharge curve at an impact speed of 47 km/h, where the capacitor was charged to 0.98 V within 39.5 ms. Based on the voltage characteristics, the harvested energy and the corresponding effective average power were calculated to be 0.23 mJ and 5.71 mW, respectively. These results highlight the harvester’s sensitivity to impact intensity as higher impact speeds lead to greater energy conversion efficiency. However, a slight decrease in energy harvesting performance was observed, which may be attributed to variations in contact surface properties during the boxing simulation, thereby affecting the energy transfer efficiency. Furthermore, the variability in applied force during boxing introduces additional challenges, necessitating a structurally robust and efficient design to ensure stable energy collection under inconsistent impact conditions. These findings suggest potential applications in wearable self-powered sensors and other portable energy harvesting systems for high-impact activities

#### 3.2.3. Stomping Test

**Figure 13:**
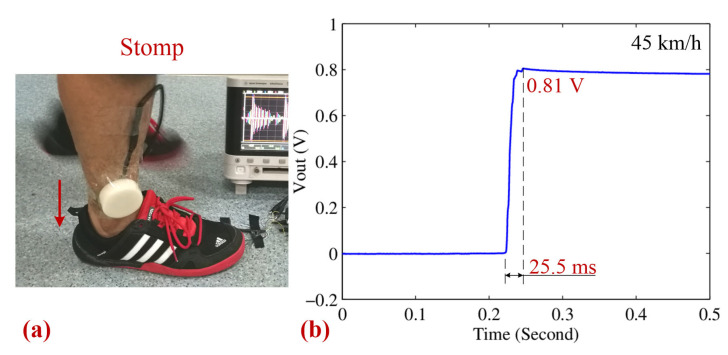
(**a**) A photograph of the stomp type. (**b**) The corresponding charging–discharging curves of a 470 μF 25 V capacitor for the stomp type.

To evaluate the performance of the motion impact energy harvester in a more stable setup, it was fixed to a shank, as shown in Figure 13a. The charging–discharging characteristics of the harvester are plotted in Figure 13b. At an impact speed of 45 km/h, the capacitor was charged to 0.81 V in 25.5 ms. The energy stored and the effective average power of the harvester were calculated to be 0.15 mJ and 6.05 mW, respectively. This test highlights the device’s potential for its integration into wearable technology or other applications that require consistent energy generation. The lower impact speed in this test resulted in reduced energy storage and power output, indicating that impact speed is a critical factor in the energy collection process. Additionally, the fixation of the harvester to the shank introduces a new variable as the method of attachment could affect the consistency of energy transfer during repeated impacts.

A sensitivity analysis of our experimental setup revealed that impact speed is a critical factor influencing the energy harvested. For example, a decrease in impact speed from 49 km/h to 45 km/h led to a reduction in both stored energy and effective average power, emphasizing the importance of optimizing operating conditions for maximum energy yield. By analyzing the voltage outputs at different impact velocities, we observed that the output increases significantly with higher impact speeds. This suggests that at higher velocities, the system transitions from rotational to oscillatory motion, resulting in greater voltage generation. Additionally, when the impact force is particularly low, the magnetic cylinder exhibits direct oscillatory behavior. The key innovation of this paper is the enhancement of the energy harvester’s output performance through multiple oscillatory motions. Throughout these tests, the harvester consistently demonstrated efficient performance, with variations influenced by the type of impact and the conditions under which they were applied. The design of the energy harvester must account for these factors to optimize energy conversion across various human motion activities. These findings have practical implications beyond the laboratory, suggesting that the harvester could be integrated into wearable technology and remote sensing applications where its ability to convert kinetic energy into electrical energy could provide a sustainable power source.

### 3.3. Efficiency Discussion and Performance Comparison

While the prototype harvester demonstrated a measurable minimum output energy of 0.15 mJ, a direct evaluation of its energy conversion efficiency requires an estimation of the mechanical input energy. Although impact force and displacement were not directly measured during clapping, stomping, and boxing tests, a reasonable approximation can be made based on typical values reported in the literature.

For instance, human clapping and hand-impact actions typically generate transient forces ranging from 20 N to 50 N, with impact displacements between 2 mm and 5 mm [39]. Taking a conservative estimate of 25 N for the average force and 3 mm for the effective displacement, the mechanical input energy can be approximated as:

(5) $$E_{in}=F\cdot d=25\;N\times 3\;\mathrm{mm}=0.075\;J$$

Given the output energy of 0.15 mJ obtained from capacitor voltage measurements, the resulting energy conversion efficiency is:

(6) $$\eta =\frac{E_{out}}{E_{in}}=\frac{0.15\mathrm{mJ}}{75\mathrm{mJ}}=0.2\%$$

This level of efficiency is comparable to those reported for passive electromagnetic energy harvesters subjected to low-frequency, impulsive mechanical forces. It confirms that the proposed design effectively converts biomechanical impact energy into electrical energy. In future work, force sensors and displacement transducers will be integrated to enable direct and precise measurement of mechanical input energy during human motion. The detailed relationship between the impact strength and the number of magnetic cylinder rotations is not analyzed in this study as the primary aim was to validate energy generation from real-world human motions. In future work, we intend to quantify this relationship under controlled impact scenarios to better evaluate the harvester’s dynamic scalability and efficiency.

### 3.4. Comparative Study

To evaluate the effectiveness of the proposed translational–rotary electromagnetic energy harvester, a performance comparison of harvesters reported in recent literature is conducted. Table 1 summarizes the comparison in terms of average power and harvested energy per impact. The proposed design achieves an average power output exceeding 3.8 mW and harvests more than 0.15 mJ per impact, which is comparable to, or even surpasses, several conventional energy harvesters that rely on vertical vibration or piezoelectric coupling under constrained input orientations. The unique dual-motion mechanism allows for repeated magnetic flux variation from a single impact event, increasing output efficiency, especially under low-frequency human activity.

## 4. Conclusions

The motion impact energy harvester introduced in this paper represents a significant advancement in biomechanical energy harvesting. The device consists of a magnetic cylinder, a magnetic ring, twelve coils, and a circular housing. It excels at capturing waste biomechanical energy, as evidenced by the damped oscillation characteristics in its output waveforms. Impact excitations, such as clapping, boxing, or stomping, can cause the magnetic cylinder to spin multiple times around the magnetic ring, demonstrating the device’s sensitivity and efficiency in energy conversion. This makes the harvester an ideal candidate for integration into wearable technology, where it could provide a sustainable power source for a range of applications, including fitness trackers and health monitoring devices.

While the prototype demonstrates the ability to charge a 470 μF capacitor to over 0.81 V within a single 39.5 ms impact event, a longer-duration scenario, such as continuous clapping or stomping at 1 Hz for 10 s, would generate approximately 1.5–2.0 mJ. This energy is sufficient to intermittently power typical low-power modules like BLE transmitters (~0.3–0.5 mJ per transmission), motion or temperature sensors, or even trigger wake-up signals in energy-aware microcontrollers (MCUs). The motion impact energy harvester also presents promising market potential for integration into motion devices, such as shoes or gloves. Furthermore, embedding it into sports equipment like footballs, basketballs, or even hockey pucks could be intriguing. Due to its structural features, the harvester is fully capable of capturing rotational and vibrational energy under the influence of gravity and magnetic forces. This versatility suggests a broader range of applications, including environments with prevalent mechanical vibrations, such as industrial settings or transportation systems, where it could power sensors and communication devices. Certainly, we will perform a more systematic uncertainty analysis with instrumented impact testing rigs and a more detailed sensitivity analysis in our future work.

## Figures and Tables

**Table 1:** Performance comparison of energy harvesters.

Reference	Mechanism	Output Energy per Impact (mJ)	Average Power (mW)	Special Features
Peralta-Braz et al. (2022) [40]	Piezoelectric	0.12	2.5	Optimized for bridge infrastructure
Kullukcu et al. (2022) [41]	Piezoelectric	0.10	0.273	Wind-driven, suitable for SHM
Kabakulak & Arslan (2021) [42]	Electromagnetic	0.2	3.1	Design for power line sensors
This work	Electromagnetc	0.15	3.8	Multi-directional impact adaptation, low-damping design

## Data Availability

The data presented in this study are available upon request from the corresponding author.
